# Posttranslational Modifications in Thyroid Cancer: Implications for Pathogenesis, Diagnosis, Classification, and Treatment

**DOI:** 10.3390/cancers14071610

**Published:** 2022-03-22

**Authors:** Jordan M. Broekhuis, Benjamin C. James, Richard D. Cummings, Per-Olof Hasselgren

**Affiliations:** 1 Department of Surgery, Beth Israel Deaconess Medical Center, Harvard Medical School, Boston, MA 02215, USA; jbroekhu@bidmc.harvard.edu (J.M.B.); bjames1@bidmc.harvard.edu (B.C.J.); rcummin1@bidmc.harvard.edu (R.D.C.); 2 Beth Israel Deaconess Medical Center, National Center for Functional Glycomics, Harvard Medical School, Boston, MA 02215, USA

**Keywords:** protein, post-translational modifications, thyroid cancer, acetylation, methylation, ubiquitination, sumoylation, glycosylation, succinylation

## Abstract

**Simple Summary:**

Alterations to human proteins following their production can result in changes to their function. In cancers specifically, these post-translational modifications (PTMs) have implications for the way in which tumors develop and progress. While most of the previous research in thyroid cancer biology has focused on gene expression, several studies have investigated PTMs and their roles in tumorigenesis, diagnosis, and treatment. Here, we review recent studies related to phosphorylation, acetylation, methylation, ubiquitination, sumoylation, glycosylation, and succinylation in human thyroid cancers. Knowledge of these various protein modifications may help to improve current diagnostics and therapeutics as well as the development of novel treatments for thyroid cancer.

**Abstract:**

There is evidence that posttranslational modifications, including phosphorylation, acetylation, methylation, ubiquitination, sumoylation, glycosylation, and succinylation, may be involved in thyroid cancer. We review recent reports supporting a role of posttranslational modifications in the tumorigenesis of thyroid cancer, sensitivity to radioiodine and other types of treatment, the identification of molecular treatment targets, and the development of molecular markers that may become useful as diagnostic tools. An increased understanding of posttranslational modifications may be an important supplement to the determination of alterations in gene expression that has gained increasing prominence in recent years.

## 1. Introduction

Although altered gene expression in thyroid cancer has been documented in a large number of studies, less attention has been paid to posttranslational modifications (PTMs). This may seem surprising because, just as changes in gene expression are important, modifications of the gene products and the roles they play in the regulation of tumor biology are equally, or maybe even more, important.

PTMs can alter the function of proteins in a multitude of ways and may be involved in the development and aggressiveness of many human cancers [1,2]. Recent studies suggest that PTMs, including phosphorylation, acetylation, methylation, ubiquitination, sumoylation, glycosylation, and succinylation, may also be involved in thyroid cancer [3,4]. An increased awareness of PTMs in thyroid cancer is important for several reasons. First, it may provide an increased understanding of the tumorigenesis involved in malignant thyroid tumors [5]. Second, it can help us understand how some advanced cancers become resistant to radioiodine and other nonsurgical treatments [6]. Third, identifying proteins and how they are modified can be helpful in finding new therapeutic targets [7]. Finally, defining PTMs in thyroid tumors may be useful in the diagnosis of thyroid nodules when cytology, imaging, and genetic testing are not sufficient to advise patients about different therapeutic options [8]. 

In the present report, we describe some of the PTMs that have been implicated in thyroid cancer. The review mainly targets physicians involved in the care of patients with thyroid tumors, including endocrinologists, endocrine surgeons, nuclear medicine specialists, pathologists, and cytologists. Scientists involved in basic research on PTMs in cancer will find more in-depth information elsewhere.

## 2. Ubiquitination

The ubiquitin system for the regulation of protein degradation was discovered more than 40 years ago [9] and has been extensively studied and reviewed in detail elsewhere [10,11]. In short, the addition of one or several ubiquitin molecules to a protein marks it for degradation by the 26S proteasome in the cytoplasm. The ubiquitination (also called ubiquitylation or ubiquitinylation) of a substrate protein is a complex and highly regulated process, in which ubiquitin is attached to one or several lysine residues of the target protein. The process is controlled by three sets of enzymes: E1s (ubiquitin activating enzymes), E2s (ubiquitin conjugating enzymes), and E3s (ubiquitin ligases). The ubiquitinated protein is inserted into the 26S proteasome and degraded into peptides that are further digested by the lysosome, ultimately resulting in free amino acids. 

In addition to ubiqutination, the removal of ubiquitin from the substrate, deubiquitination, also influences the levels of ubiquitinated proteins in the cell [12]. Deubiquitination is also a highly regulated process controlled by a set of deubiquitinating enzymes. More detailed information about the ubiquitin system is beyond the scope of the present report but has been published elsewhere [10,11].

Although the regulation of protein degradation was initially believed to be the major role of the ubiquitin system, subsequent studies have provided evidence that ubiquitination can alter several protein functions independent of proteolysis, such as protein trafficking, cellular localization, and protein–protein interactions [13].

The ubiquitination of proteins plays important roles in the biology of virtually all the cells and tissues in the body. Studies suggest that ubiquitination is involved in the tumorigenesis of many human cancers [14,15], including thyroid cancer [16]. Here, we briefly discuss some recent reports supporting the notion of a role of ubiquitination in thyroid malignant tumors.

### 2.1. Ubiquitination and Proteasomal Degradation of the Tumor Repressor PCBP1 Are Increased in Thyroid Cancer

Poly r(C)-binding protein (PCBP) 1 functions as a tumor repressor in many human cancers [17,18,19], including thyroid cancer [20]. In recent experiments, Zhang et al. [21] found evidence that the function of PCBP1 in thyroid cells reflects the abundance of the protein and that the PCBP1 levels are regulated by ubiquitin-dependent degradation. Supporting this concept, PCBP1 levels are reduced in cultured cells from thyroid cancers compared to cells from normal thyroid tissue. Additionally, degradation by the 26S proteasome of polyubuiquitinated PCBP1 was observed in thyroid cancer cells. When different ubiquitin ligases were silenced or overexpressed in cultured thyroid cells, the ubiquitination of PCBP1 was regulated by the ubiquitin ligase UBE4A. Taken together, the results reported by Zhang et al. [21] support a model in which increased UBE4A activity resulted in the ubiquitination and destruction of the tumor suppressor PCBP1 in thyroid cancer.

Importantly, when in the same report [21] studies were performed on tumor and normal adjacent thyroid tissue from patients undergoing thyroidectomy for cancer, some patients had particularly high levels of UBE4A combined with reduced PCBP1 levels in the cancer; these patients had a less favorable clinical outcome than the patients with less pronounced changes in UBE4A and PCBP1 levels. From their observations, the authors concluded that UBE4A-regulated ubiquitination and degradation of PCBP1 may be involved in the tumorigenesis of thyroid cancer. They also proposed that UBE4A may be considered a tumor promoter in thyroid cancer, the levels and activity of which may be used as a prognostic factor for patients with thyroid cancer.

### 2.2. The Activity of the Ubiquitin Ligase Smurf1 and Ubiquitin-Dependent Degradation of the Tumor Suppressor Kisspeptin-1 Are Increased in Thyroid Cancer

Further evidence for the role of protein ubiquitination in thyroid cancer was reported by Yan et al. [22]. In recent experiments, they found that the expression of the ubiquitin ligase Smad ubiquitination regulatory factor 1 (Smurf1) was increased in tumor tissue compared with normal adjacent thyroid tissue in patients undergoing thyroidectomy for cancer. A similar pattern was observed in human thyroid cancer cell lines.

In additional experiments, the overexpression of Smurf1 in cultured thyrocytes stimulated migration, invasion, and proliferation of the cells, suggesting that Smurf1 may be involved in thyroid cancer aggressiveness. Because previous studies suggested that the expression of the tumor suppressor Kisspeptin-1 was downregulated in thyroid cancer [23,24] and that the downregulation or absence of Kisspeptin-1 was involved in thyroid tumorigenesis, invasion, and metastasis [25], the authors next examined whether the increased Smurf1 expression may be involved in downregulated Kisspeptin-1 expression.

When cultured thyroid cancer cells were transfected with Smurf1, Kisspetin-1 protein levels declined in a dose-dependent fashion. In the same cells, the ubiquitination of Kisspeptin-1 increased, and when cells were treated with the proteasome inhibitor MG-132, the levels of ubiquitinated Kisspeptin-1 increased substantially. Taken together, these results suggest that Smurf1 ubiquitinates Kisspeptin-1, leading to its proteasome-dependent degradation. In additional experiments, the results showed that the overexpression of Kisspeptin-1 inhibited the migration, invasion, and proliferation of cultured thyroid cancer cells, lending support to the notion of the role of Kisspeptin-1 as a tumor repressor in thyroid cancer.

Overall, the results reported by Yan et al. [22] support a model in which the development of thyroid cancer may at least in part be regulated by Smurf1-induced ubiquitination and the degradation of the tumor suppressor Kisspeptin-1.

### 2.3. Ubiquitin-Dependent Degradation of VEGFR2 Decreases Angiogenesis and Aggressiveness of Poorly Differentiated PTC

Although the studies described above suggest that the ubiquitination and degradation of some proteins (PCBP1 and Kisspeptin-1) may increase the aggressiveness of thyroid cancer, the same molecular mechanisms may reduce the aggressive features of thyroid cancers under certain circumstances. The opposite effects of ubiquitination depend upon which specific protein(s) are ubiquitinated and degraded. When tumor repressors are degraded it is, of course, expected to that increased tumor aggressiveness will be observed, whereas the opposite is expected if a protein acting as a tumor promoter is ubiquitinated and destructed. 

Angiogenesis is increased in many human cancers [26,27], in part reflecting the increased expression of the VEGF receptor 2 (VEGFR2). VEGFR2 is the main receptor transmitting VEGF signals and many cancers, including thyroid cancer, have elevated levels of VEGF2R [28,29,30]. VEGFR2 promotes angiogenesis and tumor growth by stimulating endothelial cell proliferation and migration. 

Shaik et al. [31] examined the mechanisms that may regulate VEGFR2 expression in thyroid cancer. In experiments performed in a human thyroid cancer cell line, the cellular abundance of VEGFR2 was regulated by its degradation. Additional experiments showed that the degradation of VEGFR2 was regulated by its ubiquitination and that the ubiquitination in turn was governed by the E3 ligase Skip1-Cullin1-F-box [SCF(β-TRCP)].

Because the ubiquitination of proteins may be influenced by phosphorylation and because the multi-tyrosine kinase inhibitor sorafenib has been used in the treatment of aggressive thyroid cancers [32], experiments were performed to examine whether a kinase may be involved in the ubiquitination of VEGFR2 in thyroid cancer cells [31]. By using different kinase inhibitors, it was found that the SCF(β-TRCP)–ubiquitin-dependent degradation of VEGFR2 was regulated by casein-kinase1 (CSK1). Together, the results suggested that the expression of VEGFR2 in human thyroid cancer cells is regulated by a CSK1-SCF(β-TRCP)-ubiquitin-dependent mechanism.

In order to test whether the manipulation of VEGFR2 levels has tumorigenic consequences, Shaik et al. [31] performed additional experiments in which angiogenesis, cell migration, and invasiveness were assessed in a complex set of in vitro experiments. The results from these experiments indicated that CSK1-SCF(β-TRCP)-ubiquitin-dependent degradation of VEGFR2 decreases angiogenesis and endothelial and thyroid cancer cell migration and invasiveness.

Studies have shown that the tyrosine kinase inhibitor sorafenib may induce only a partial response in some patients with aggressive and metastatic forms of thyroid cancer [32,33]. In order to examine whether sensitivity to sorafenib may correlate with VEGFR2 levels in thyroid cancer cells, Shaik et al. [31] treated cultured human cancer cells expressing different levels of VEGFR2 with different concentrations of sorafenib. The results indicated that cells with high VEGFR2 levels had increased sensitivity to sorafenib. Based on these observations, the authors speculated that thyroid cancer patients with low tumor levels of β-TRCP (resulting in high levels of VEGFR2) may respond more favorably to treatment with sorafenib, and perhaps other tyrosine kinase inhibitors as well.

Overall, the results in the study by Shaik et al. [31] suggest that reduced CSK1-SCF β-TRCP-dependent ubiquitination may lead to high VEGFR2 levels and angiogenesis in thyroid cancer. In addition, the results suggest that the improved response to sorafenib treatment seen in some patients may reflect high VEGFR2 expression and that the expression of VEGFR2 may be used as a biomarker that may help select patients for treatment with sorafenib.

It should be noted that although the elegant in vitro experiments performed by Shaik et al. [31] support the concept that ubiquitin-dependent degradation of VEGFR2 decreases the aggressiveness of thyroid cancer by suppressing angiogenesis and the migration of the cancer cells, it will be important in future studies to determine whether the same molecular and cellular mechanisms are involved in patients with thyroid cancer.

### 2.4. Ubiquitin Staining May Help Differentiate PTC from NIFTP

In recent years, it has become clear that the noninvasive follicular variant of thyroid neoplasm with papillary-like nuclear features (NIFTP) clinically behaves like a non-malignant tumor and that thyroid lobectomy, rather than total thyroidectomy, is sufficient in most patients with this diagnosis [34]. Therefore, studies have been performed recently in attempts to identify methods to diagnose NIFTP preoperatively, which would help counsel patients regarding the extent of surgery, particularly when presenting with a thyroid nodule and fine-needle aspiration cytology suspicious for malignancy [35,36]. Although certain cytomorphological and ultrasonographic features, as well as genetic alterations, may raise the suspicion that a thyroid nodule harbors a NIFTP rather than a cancer [37,38], none of the characteristics examined so far have been able to discriminate between NIFTP and PTC with high accuracy.

One of the cytomorphological features that have been used to differentiate NIFTP from PTC is the presence of nuclear pseudoinclusions [34]. True nuclear pseudoinclusions represent redundant nuclear membrane present in malignant thyroid cells, similar to the mechanism of the formation of nuclear grooves in PTC [39]. A true nuclear pseudoinclusion, therefore, contains cytoplasm that in its composition matches the cytoplasm peripheral to the nucleus. The pseudoinclusions are typically surrounded by a clear, sharp nuclear membrane and are usually present only in a few cells in PTC and limited to one per nucleus. This is different from artifactual pseudo-pseudoinclusions, which are often multiple per nucleus and poorly demarcated, and frequently occur in a high percentage of cells [40].

The quality and frequency of pseudoinclusions required for a diagnosis of NIFTP are unclear. Initially, they were described as “few” [34,41], but more recent stringent criteria do not allow for any [42,43]. The confusion with regards to the presence of pseudoinclusions for the diagnosis of NIFTP may also reflect some of the difficulties in differentiating true pseudoinclusions from pseudo-pseudoinclusions.

In a recent study by Cracolici et al. [44], the interesting observation was made that the cytoplasm in true nuclear pseudoinclusions is always positive for ubiquitin immunostaining, whereas pseudo-pseudoinclusions do not exhibit ubiquitin immunostaining. From these observations, the authors proposed that ubiquitin immunostaining may be used to differentiate true pseudoinclusions from pseudo-pseudoinclusions and that, when using this criterion for true pseudoinclusions, their presence may be used to exclude NIFTP.

Although the study by Cracolici et al. [44] did not define which cytoplasmic proteins in the pseudoinclusions were ubiquitinated or the function and biological role of the ubiquitinated proteins, the observations may be of clinical importance in helping differentiate NIFTP from PTC. This would be particularly helpful in patients presenting with a Bethesda V or VI nodule and in whom the extent of surgery (i.e., lobectomy vs. total thyroidectomy) may be influenced by the degree of suspicion that the nodule is NIFTP rather than cancer. It will be important in future studies to determine whether ubiquitin immunostaining can be used in cytological specimens after the fine needle aspiration of thyroid nodules to make the diagnosis of NIFTP. The potential to diagnose NIFTP preoperatively may prevent the overtreatment of some patients who have cytology that is suspicious for cancer (Bethesda V), a cytology classification that has commonly prompted total upfront thyroidectomy in many patients [35,36,45].

## 3. Sumoylation

Small ubiquitin-like modifier (SUMO) proteins resemble ubiquitin. Humans have four SUMO isoforms, SUMO 1–4. Sumoylation, the enzymatic attachment of a SUMO molecule to one or several lysine residues of a target protein, results in posttranslational modification that influences protein stability, cellular localization (protein trafficking), and the function of transcription factors. In contrast to ubiquitin, SUMO does not mark proteins for degradation. Protein sumoylation and its role in human disease were reviewed recently [46].

The sumoylation of proteins is regulated in a similar fashion to ubiquitination by three distinct sets of enzymes: E1 enzymes activate SUMO; E2 enzymes are SUMO-conjugating enzymes; and E3 enzymes are SUMO ligases, regulating the actual attachment of SUMO to the target protein. As with the ubiquitin system, SUMO can be removed from the substrate by a set of desumoylation enzymes.

There is evidence that sumoylation may be involved in the tumorigenesis of various cancers [47], including thyroid cancer [48,49,50,51]. Here, we review studies providing evidence for the role of sumoylation in thyroid cancer. 

### 3.1. Decreased Nuclear Levels of Sumoylated PDGF-C in Thyroid Cancer

Platelet-derived growth factor-C (PDGF-C) belongs to a superfamily of growth factors that are involved in the stimulation of the growth, angiogenesis, and tumorigenesis of various cancers [52,53]. In recent studies, it was reported that the nuclear levels of sumoylated PDGF-C were reduced in thyroid cancer cells as compared with normal thyroid cells [48]. The results were interpreted as being consistent with inhibited sumoylation of the growth factor in thyroid cancer, although increased desumoylation may have been involved as well. Regardless, the researchers speculated that the reduced levels of sumoylated PDGF-C may be involved in the tumorigenesis in thyroid cancer.

### 3.2. Sumoylation Inhibits the Tumor Suppressor CCDC6 in Thyroid Cancer Cells by Reducing Its Interaction with the Transcription Factor CREB-1

CREB is a transcription factor involved in many cellular responses, such as proliferation, survival, and differentiation. After nuclear translocation, the 65 kDa phosphoprotein CCDC6 interacts with and inhibits the transcription factor CREB-1, resulting in transcriptional repression. CCDC6 may therefore act as a tumor repressor [54].

In recent studies, evidence was found that the tumor-suppressive (pro-apoptotic) effects of CCDC6 were influenced by sumoylation [50]. The attachment of SUMO2 to CCDC6 resulted in the sequestration of CCDC6 in the cytoplasm of cultured thyrocytes and a downregulation of its interaction with CREB-1. Therefore, the sumoylation of CCDC6 resulted in the reduced inhibition of CREB-1 allowing for increased CREB-1 dependent transcriptional activity and cellular proliferation. Taken together, the results indicate that the sumoylation of the tumor repressor CCDC6 may be a factor involved in thyroid cancer, providing an additional mechanism of sustained neoplastic growth.

### 3.3. SUMO Inhibitors May Offer a Novel Approach to the Treatment of Anaplastic Thyroid Cancer

Anaplastic thyroid cancer is one of the most aggressive human cancers. Although it is rare, accounting for only 1–2% of thyroid cancers, it causes 30–50% of all thyroid cancer-specific deaths [55]. 

The results from recent experiments in anaplastic thyroid cancer cell lines suggest that the dedifferentiation of papillary thyroid cancer into anaplastic thyroid cancer is caused at least in part by the sumoylation of the transcription factor TFAP2A [49]. The sumoylation of TFAP2A results in altered patterns of gene expression associated with anaplastic thyroid cancer. 

As an extension of their experiments in cultured anaplastic thyroid cancer cells, the authors also tested the effects of two SUMO inhibitors, PYR-41 and anacardic acid, in mice harboring a tumor mimicking anaplastic thyroid cancer [49]. Treatment with SUMO inhibitors reduced tumor size and significantly improved tumor-free survival among the tumor-bearing mice. Although the results from these experiments need to be interpreted with caution, since they were generated in cultured cell lines and mice, they point towards the possibility that the outcome in patients with anaplastic thyroid cancer may be improved by inhibiting the PTM of a transcription factor involved in the pathogenesis of this dreadful disease.

## 4. Acetylation

Protein acetylation is regulated by histone acetyltransferase (HAT) and histone deacetylase (HDAC) activities. The HAT and HDAC enzymes catalyze the acetylation and deacetylation, respectively, of lysine residues in histones as well as other proteins. The acetylation and deacetylation of histones play important roles in gene transcription and are the most widely studied aspects of these posttranslational modifications [56,57]. Histones are the main protein components of chromatin and serve as the “spools” around which DNA is organized. Histone acetylation transforms the chromatin into a more “relaxed” configuration, promoting the increased access of transcription factors to DNA and increased gene transcription. By contrast, deacetylation results in transcriptional deactivation (gene silencing) by wrapping DNA more tightly around the histone and decreasing the access of transcription factors to the DNA. 

In addition to histones, the function of many other cellular proteins is also influenced by acetylation and deacetylation [58]. Acetylation may control the functions of proteins either directly or indirectly by affecting the stability of the proteins. The acetylation of transcription factors and nuclear cofactors may regulate transcriptional activity independently of histone acetylation [59]. 

The regulation of acetylation and deacetylation is complex, with multiple classes of HATs and HDACs having been described [60]. Members of the different classes of HATs and HDACs perform specific functions by targeting different histones and other proteins and by binding to different lysine residues. Previous reports suggest that protein acetylation is involved in many human diseases, including inflammatory diseases [61], heart diseases [62,63], muscle wasting [64,65], and neurological disorders [66]. Importantly, there is evidence that acetylation and deacetylation are involved in multiple human cancers [67,68], including thyroid cancer [69,70,71]. 

### 4.1. Histone Acetylation Is Differentially Regulated in Different Types of Thyroid Tumor

In a recent study, Puppin et al. [69] examined the levels of acetylated histones in different thyroid tumors (follicular adenoma, PTC, follicular thyroid cancer (FTC), and undifferentiated thyroid cancer) and in normal thyroid tissue from patients undergoing thyroidectomy. Antibodies specific for acetylated lysine residues in H3 and H4 histones were used for immunohistochemistry and the levels of acetylated histones were determined by using a semi-quantitative scoring system. The authors found that the levels of acetylation were specific for different lysine residues in the different histones and varied between the tumors. For example, the levels of H3 acetylated at lysine residues 9–14 were increased in all types of tumor compared with the levels in normal thyroid tissue, whereas the acetylation of lysine residue 18 in the H3 histone was increased in follicular adenomas, PTC, and FTC, but not in undifferentiated thyroid cancer. The level of acetylated lysine 12 in the H4 histone was increased in follicular adenomas but was unchanged in PTC, FTC, and undifferentiated thyroid cancer.

The observations reported by Puppin et al. [69] are important because they support the concept that changes in the levels of acetylated histones may be involved in the tumorigenesis of thyroid tumors. It will be important in future studies to determine whether similar specific profiles of acetylated histones in different thyroid tumors may be identifiable in fine needle aspiration cytology specimens increasing the accuracy of the diagnosis of thyroid nodules without the need for surgery and histopathology.

### 4.2. Treatment of Thyroid Cancer with HDAC and HAT Inhibitors

In a recent report by Russo et al. [70], preclinical studies of HDAC inhibitors in cultured thyroid cancer cells and studies in patients with thyroid cancer were reviewed. In these reports, drugs that inhibit multiple classes of HDACs, as well as more specific HDAC inhibitors, were examined. The studies were important in light of other accounts indicating that HDAC activity is increased in various types of thyroid cancer [72].

Experiments in cultured thyroid cancer cells indicate that the inhibitors of HDACs can exert anti-proliferative and re-differentiating effects in poorly differentiated cancers, suggesting that HDACs may be potential molecular targets in the treatment of thyroid cancer. However, in the review by Russo et al. [70], the authors concluded that HDAC inhibitors “are emerging as very promising drugs for this purpose,” and that controlled clinical studies are needed to support a role of HDAC inhibitors in patients with thyroid cancer and who need more advanced treatment in addition to surgery and radioiodine.

Because the levels of acetylated histones and other proteins are influenced by both HDACs and HATs, other studies have focused on the effects of HAT inhibitors [73]. Several drugs can inhibit HAT activity, including curcumin, garcinol, anacardic acid, and bisubstrate inhibitors. Although preclinical studies of these compounds have provided promising results, future studies will have to determine the potential value of HAT inhibitors in the treatment of patients with advanced thyroid cancer.

The fact that both HDAC inhibitors (increasing levels of acetylated proteins) and HAT inhibitors (decreasing levels of acetylated proteins) are being pursued as potential drugs to treat thyroid cancer reflects the complexity of the mechanisms involved in the regulation of the acetylation and deacetylation of histones and other proteins in thyroid cancer. It will probably take many years to definitively answer the question of whether manipulating the levels of acetylated histones and other proteins may be useful in the treatment of thyroid cancer.

## 5. Phosphorylation

Phosphorylation (attachment of a phosphoryl group to amino acids within a protein) is one of the most extensively studied posttranslational modifications. It has been calculated that about a third of all the proteins in eukaryotes are phosphorylated [74], illustrating the huge impact of this posttranslational modification can have in cell biology.

Phosphorylation has far-reaching effects on cellular functions, not only because it can influence the stability and function of the target protein itself, but also because it can regulate and interact with other posttranslational modifications, allowing multiple layers of complexity in the regulation of cellular function. Phosphorylation is involved in the regulation of gene expression, protein stability, and protein–protein interactions. Recent studies have demonstrated that the perturbed phosphorylation of proteins plays important roles in cancer development, at least in part reflecting changes in cell growth and differentiation [75,76].

Protein phosphorylation is regulated by kinases, transcribed by over 500 kinase-encoding genes, and by nearly 200 phosphatase-encoding genes, which dephosphorylate phosphoproteins. The cellular levels of phosphorylated proteins thus reflect the balance between kinase and phosphatase activities [77]. Although nine different amino acids may be phosphorylated, the most commonly phosphorylated are threonine, serine, and tyrosine. Among different kinases, tyrosine kinases play the most important role in different types of cancer, including thyroid cancer [76]. 

The complexity of protein phosphorylation is illustrated by the fact that multiple sites in a protein can be phosphorylated by the same kinase or by multiple kinases. In addition, proteins can undergo multiple posttranslational modifications regulated by the interaction between different mechanisms, for example phosphorylation and ubiquitination, and phosphorylation and glycosylation (addition of O-linked *N*-acetylglucosamine (GlcNAc) to Ser/Thr residues) [78].

### 5.1. The MAPK and PI3K-Akt Signaling Pathways in Thyroid Cancer

**Figure 1:**
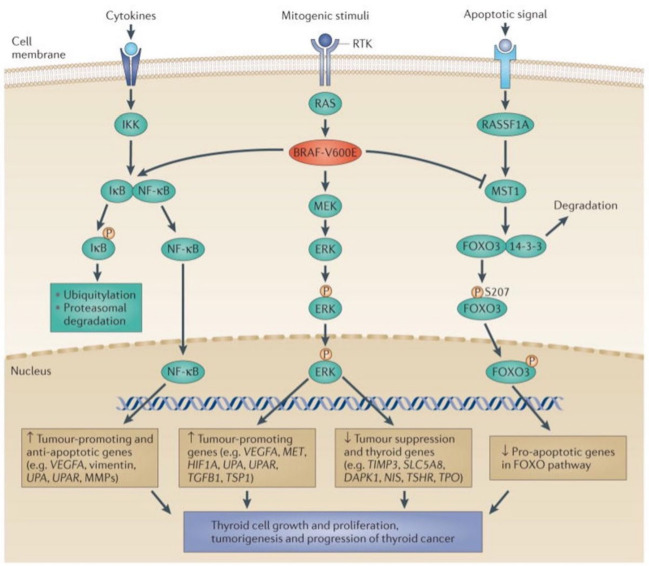
The MAPK and related pathways in thyroid cancer. Shown in the (**middle**) of the figure is the classical MAPK pathway leading from an extracellular mitogenic stimulus that activates a receptor tyrosine kinase (RTK) in the cell membrane, to RAS, RAF (shown as BRAF-V600E), MEK and ERK. ERK enters the nucleus where it upregulates tumor-promoting genes and downregulates tumor suppressor genes and thyroid iodide-handling genes. On the (**left**) side of the figure is the nuclear factor-κB (NF-κB) pathway, in which extracellular stimuli activate the pathway, leading to activation of the inhibitor of κB (IκB) kinase (IKK), resulting in the phosphorylation of IκB. IκB becomes dissociated from NF-κB and then enters the nucleus to promote the expression of tumor-promoting genes. Through an undefined mechanism, BRAF-V600E promotes the phosphorylation of IκB and the release of NF-κB, thus activating the NF-κB pathway. Shown on the (**right**) side of the figure is the RASSF1–mammalian STE20-like protein kinase 1 (MST1)–forkhead box O3 (FOXO3) pathway. Activated by extracellular pro-apoptotic stimuli, RASSF1A activates MST1which phosphorylates FOXO3. The resulting phosphorylated FOXO3 becomes dissociated from 14-3-3 proteins and enters the nucleus to promote the expression of pro-apoptotic genes in the FOXO pathway. BRAF-V600E directly inhibits MST1 and prevents its activation by RASSF1A, thereby suppressing the pro-apoptotic signaling of the FOXO3 pathway. The downward arrow for the FOXO activities shown in the nucleus indicates this negative effect of BRAF-V600E on pro-apoptotic genes. DAPK1, death-associated protein kinase 1; HIF1A, hypoxia-inducible factor 1α; MMP, matrix metalloproteinase; NIS, sodium–iodide symporter; TGFB1, transforming growth factor β1; TIMP3, tissue inhibitor of metalloproteinases 3; TPO, thyroid peroxidase; TSHR, thyroid-stimulating hormone receptor; TSP1, thrombospondin 1; UPA, urokinase plasminogen activator; UPAR, urokinase plasminogen activator receptor; VEGFA, vascular endothelial growth factor A. Figure reprinted from Ref. [79], by permission.

The role of the mitogen-activated protein kinase (MAPK) signaling pathway in thyroid tumorigenesis is well established [79]. In addition to mitogens, the pathway can also be regulated by cytokines and apoptotic signaling. The mechanisms involved in the MAPK signaling pathway and its influence on thyroid tumorigenesis are summarized in Figure 1. 

In short, the activation of BRAF-V600E results in downstream phosphorylation and activation of ERK (extracellular signal-regulated kinase) which, after nuclear translocation, upregulates tumor-promoting genes and downregulates tumor-suppressor genes. The activation of BRAF-V600E also results in phosphorylation of the inhibitor of kappa B (IκB) leading to its ubiquitin-proteasome-dependent degradation and the release of NF-κB that can now translocate to the nucleus and upregulate tumor-promoting and anti-apoptotic genes. Concomitantly, BRAF-V600E inhibits the phosphorylation of the transcription factor FOXO3, resulting in the reduced pro-apoptotic activity of FOXO3. 

Taken together, the mechanisms described in Figure 1 act in concert to stimulate thyroid cell growth and proliferation, tumorigenesis, and the progression of thyroid cancer.

**Figure 2:**
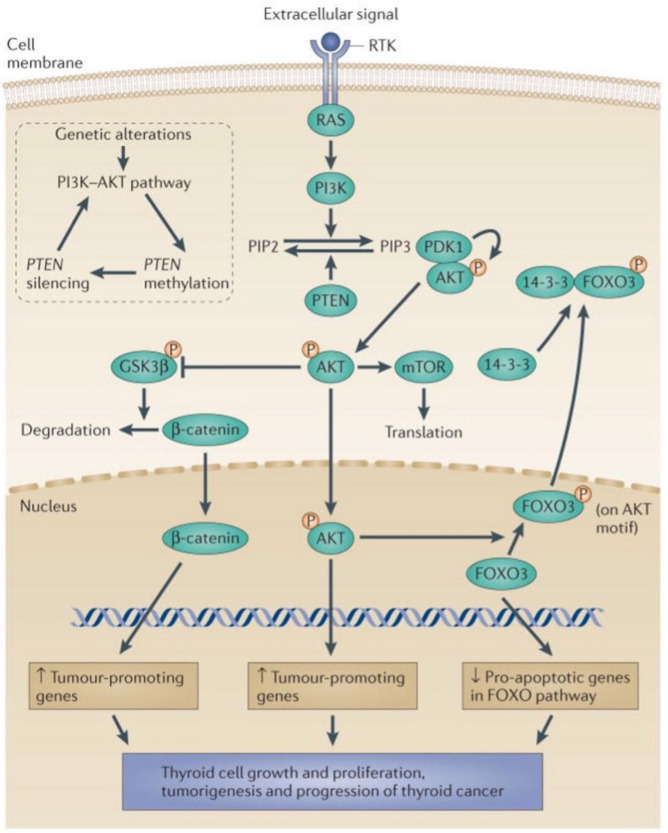
The PI3K-AKT and related pathways in thyroid cancer. Extracellular signals activate receptor tyrosine kinases (RTKs), leading to the activation of RAS and PI3K. Activated PI3K catalyzes the conversion of phosphatidylinositol (4,5)-bisphosphate (PIP2) to phosphatidylinositol (3,4,5)-trisphosphate (PIP3). PIP3 activates 3-phosphoinositide-dependent protein kinase 1 (PDK1; also known as PDPK1), which consequently associates with AKT and leads to phosphorylation (P) and the activation of AKT by PDK1. Phosphorylated AKT enters the nucleus, where it induces tumor-promoting genes. In the cytoplasm, phospho-AKT also activates other signaling molecules or pathways, including the mTOR pathway, which has an important role in tumorigenesis. Phospho-AKT can also directly phosphorylate glycogen synthase kinase 3β (GSK3β), relieving the GSK3β-mediated suppression of β-catenin. Consequently, β-catenin can enter the nucleus, where it promotes the expression of tumor-promoting genes. In the nucleus, phospho-AKT can phosphorylate forkhead box O3 (FOXO3) on its AKT-specific motif. This phosphorylated FOXO3 is translocated out of the nucleus to the cytoplasm, where it binds 14-3-3 proteins to be sequestered in the cytoplasm, thus terminating the pro-apoptotic activities of the FOXO3 pathway. The downward arrow for the FOXO activities in the nucleus in the figure indicates this negative effect of AKT on pro-apoptotic genes in the FOXO pathway, which would otherwise be upregulated by the FOXO3 pathway. The major negative regulatory mechanism of the PI3K–AKT pathway is PTEN, which is a phosphatase that converts PIP3 to PIP2, thus terminating the activation of the pathway. The inset shows the self-enhancement mechanism of PI3K–AKT signaling, resulting in a loss of termination of the signaling. Figure reprinted from Ref. [79], by permission.

The phosphorylation and activation of protein kinase B (Akt) is an additional important mechanism of thyroid tumorigenesis [3,79,80]. The most common mechanism of Akt phosphorylation is the one regulated by phosphatidylinositol 3-kinase (PI3K). The mechanisms of the PI3K-Akt signaling pathway in thyroid tumorigenesis are summarized in Figure 2. Phosphorylated Akt translocates to the nucleus and activates tumor-promoting genes. While still in the cytoplasm, phosphorylated Akt can also increase the phosphorylation of GSK-3β, which allows β-catenin to enter the nucleus and upregulate tumor-promoting genes. Finally, nuclear phosphorylated Akt can phosphorylate FOXO3, resulting in its export out of the nucleus and a decrease in its downregulation of proapoptotic genes. Thus, the activation of the PI3K-Akt signaling pathway stimulates thyroid tumorigenesis through at least three pathways, i.e., the nuclear translocation of β-catenin and phosphorylated Akt and the expulsion of phosphorylated FOXO3 from the nucleus.

An awareness of phosphorylation in thyroid cancer is important not only because it may help us better understand the tumorigenesis of these tumors, but also because it may help define targets for small molecule inhibitors that may be useful in the treatment of certain types of thyroid cancer. For example, the use of kinase inhibitors in the treatment of aggressive thyroid cancers has attracted great interest in recent years [81].

Given the complexity of the mechanisms involved in the phosphorylation in thyroid cancer and the large number of publications in the field, only a few examples of studies providing evidence for a role of phosphorylation in thyroid cancer are discussed here. Some of the reports illustrate the role of phosphorylation in the tumorigenesis, whereas other studies reflect therapeutic applications involving the use of kinase inhibitors. Although the size of the research area covering the role of protein phosphorylation in thyroid cancer prevents a more complete review, the examples provided here support the overall argument of the current review that PTMs play important roles in the biology of thyroid tumors and may offer targets for treatment.

### 5.2. Phosphorylation and Activation of Akt by Downregulated Expression of the Transcription Factor ZNF677 Is Involved in Thyroid Tumorigenesis

Although increased Akt phosphorylation and activity in thyroid cancer most often reflects stimulated PI3K-Akt signaling, other mechanisms may also be involved. For example, in a recent study by Li et al. [82], evidence was found that Akt phosphorylation in human thyroid cancer was caused by the decreased expression of the transcription factor zinc finger protein ZNF677. Previous studies had revealed that reduced levels of zinc finger proteins were associated with cancer progression [83]. In the report by Li et al. [82], the expression of ZNF677 was reduced in thyroid cancer tissue from patients undergoing surgery for papillary thyroid cancer compared with nonmalignant thyroid tissue from the same patients. Similar observations were made in cultured human thyroid cancer cells. Additional results suggested that the reduced abundance of ZNF677 in thyroid cancer was caused by methylation-induced inhibition of the promoter in the ZNF677 gene.

Further support for a role of ZNF677 in Akt phosphorylation was generated in experiments in which ZNF677 was overexpressed in cultured thyroid cancer cells. In these experiments, high levels of ZNF677 reduced the phosphorylation of Akt and, importantly, inhibited cell proliferation. The results indicate that the transcription factor ZNF677 acts as a tumor suppressor secondary to its inhibitory effect on Akt phosphorylation. The findings therefore add to the “treasure trove” of small molecules that may be targeted for the treatment of thyroid cancer [84].

### 5.3. Glycosylation and Phosphorylation of AkT Promote Thyroid Malignancy

In addition to phosphorylation, Akt1 activity is also partly regulated by the addition of O-GlcNAc to its Ser/Thr residues. The addition of O-GlcNAc occurs in the cytoplasm and nucleus, and occurs through the action of O-GlcNActransferase (OGT), whereas the removal of O-GlcNAc, akin to dephosphorylation, takes place through the action of O-GlcNAcase (OGA) [78]. Remarkably, these O-GlcNAcylated glycoproteins include all known phosphorylated proteins and are thus a wide-spread form of PTM among cytoplasmic and nuclear proteins.

Zhang et al. [85] found that Akt1 activity was stimulated by elevated O-GlcNAcylation by enhanced Akt1 phosphorylation at Ser473, indicating that O-GlcNAcylation can directly influence phosphorylation. As O-GlcNAcylation is reversible, the inhibition of the OGA through the drug Thiamet-G, which results in elevated steady-state O-GlcNAcylation, enhanced the invasion in vitro of human thyroid anaplastic cancer 8305C cells. These results are consistent with prior studies showing that the downregulation of OGA results in the activation of Akt1 in thyroid anaplastic cells [86] and that O-GlcNAcylation enhances anaplastic thyroid carcinoma malignancy [87]. Thus, phosphorylation and O-GlcNAcylation together may regulate PI3K/Akt signaling, and drugs targeted to affect this pathway may be useful for the treatment of thyroid anaplastic cancer.

In relation to the O-GlcNAcylation regulation of Akt1, another key pathway of thyroid tumor growth is regulated by Yes-associated protein (YAP), which is a core component of the Hippo pathway, and is a tumor suppressor. The activity of YAP is also regulated by O-GlcNAcylation and phosphorylation [88]. When the Hippo pathway is upregulated, the phosphorylation of macrophage-stimulating 1/2 (MST1/2) is triggered, thereby inactivating YAP, whereas the downregulation of Hippo leads to YAP shuttling into the nucleus and acting as a transcriptional co-activator of the TEAD family of transcription factors, which stimulates growth and cellular proliferation. Li et al. [88] demonstrated that the O-GlcNAcylation of Ser109 on YAP induced YAP Ser127 dephosphorylation. The factors regulating O-GlcNAcylation in this pathway are poorly understood, but understanding this pathway and the roles of YAP in thyroid cancer could offer further insight.

### 5.4. Deacetylation and Phosphorylation of Akt Promotes Thyroid Tumorigenesis, Illustrating the Role of Interactions between Different Posttranslational Modifications

Based on previous observations that the histone deacetylase SIRT7 is upregulated in thyroid cancer [89,90], and given the established role of Akt in thyroid tumorigenesis [79,80,91], Li et al. [5] recently investigated whether SIRT7 stimulates thyroid cancer proliferation and invasiveness by activating Akt. Through a series of experiments utilizing tissue from patients undergoing thyroidectomy for PTC, cultured human thyroid cancer cell lines, and thyroid cancer xenografts in nude mice, the authors confirmed previous reports of increased expression of SIRT7 in thyroid cancer. Additional experiments provided evidence that high levels of SIRT7 exerted stimulated cellular proliferation, invasiveness, and tumor growth in mice carrying implanted human thyroid cancer secondary to the deacetylation of Akt followed by the phosphorylation of the kinase. In addition to Akt, the ribosomal protein S kinase beta-1 (p70S6K1) was also phosphorylated by the action of SIRT7, albeit through a slightly different mechanism.

The results reported by Li et al. [5] are important because they illustrate the complexity of the mechanisms involved in thyroid tumorigenesis, with different types of posttranslational modifications interacting with each other. The study suggests that SIRT7 may be targeted in the treatment of aggressive thyroid cancers.

### 5.5. Phosphorylation of the Tumor Suppressor RB (Retinoblastoma Protein) Is Involved in Anaplastic Thyroid Cancer

Although certain novel therapeutic advances have shown promise, the outcome in patients with anaplastic thyroid cancer remains dismal [92,93]. Much current research is focused on a better understanding of the mechanisms involved in the tumorigenesis of anaplastic thyroid cancer, with the ultimate goal of finding molecular targets for treatment. 

Recent studies suggest that the increased phosphorylation of the tumor suppressor RB (retinoblastoma protein) may be involved in the development of anaplastic thyroid cancer [94]. The phosphorylation of RB results in its inactivation, promoting cellular proliferation and malignant transformation [95]. The phosphorylation of RB is regulated by the cyclin-dependent kinases, CDK4 and CDK6 [95]. Given these observations, studies have examined whether the inhibition of the CDK-dependent phosphorylation of RB may have beneficial therapeutic effects in anaplastic thyroid cancer. Indeed, recent reports in which selective inhibitors of cyclin-dependent kinases inhibited cell proliferation and the growth of experimental anaplastic thyroid cancer in rodents supported the notion that these drugs may become useful in the treatment of this aggressive tumor [94,96].

Wong et al. [94] tested the effects of the CDK4/6 inhibitor palbociclib in cell lines from anaplastic thyroid cancers and in mice carrying a xenograft of human anaplastic thyroid cancer. When cultured anaplastic thyroid cancer cells were treated with the kinase inhibitor, RB phosphorylation and cellular proliferation were inhibited. When mice carrying the anaplastic thyroid xenograft were treated with the drug, a dramatic inhibition of tumor growth was noticed. One problem, however, was that the cells in vitro and the tumors in vivo became resistant to treatment with palbociclib, similar to previous observations during treatment for breast cancer [97]. Additional experiments reported by Wong et al. [95] provided evidence that the development of resistance in anaplastic thyroid cancer was at least in part caused by the increased expression of cyclin D3 and upregulated CDK4/6 activity. 

Because the increase in cyclin D3 expression and CDK4/6 activity was prevented by the PI3K/mTOR inhibitor omipalisib, the investigators then tested the effects of combined treatment with both inhibitors. The treatment of mice carrying a xenograft of human anaplastic thyroid cancer with a combination of palbociclib and omipalisib resulted in a long-lasting and complete inhibition of tumor growth. In addition, the combination treatment arrested further growth if the tumor was allowed to develop to a more advanced stage before the treatment was started. This was an important observation considering that patients with anaplastic thyroid cancer often present when the tumor has already reached an advanced stage, with local invasion of surrounding structures.

Taken together, the results reported by Wong et al. [94] support an important role of CDK4/6-dependent phosphorylation of the tumor suppressor RB in anaplastic thyroid cancer and that the inhibition of this phosphorylation can block the growth of this challenging tumor. Because the results were observed in cultured anaplastic thyroid cancer cells and in mice carrying anaplastic thyroid cancer xenografts, the observations need to be interpreted with caution. The effects of CDK4/6 inhibitors in patients with anaplastic thyroid cancer will have to await the outcome of clinical trials. The observations, however, are important because they support the role of phosphorylation as an important factor in the development of anaplastic thyroid cancer.

## 6. Methylation

**Figure 3:**
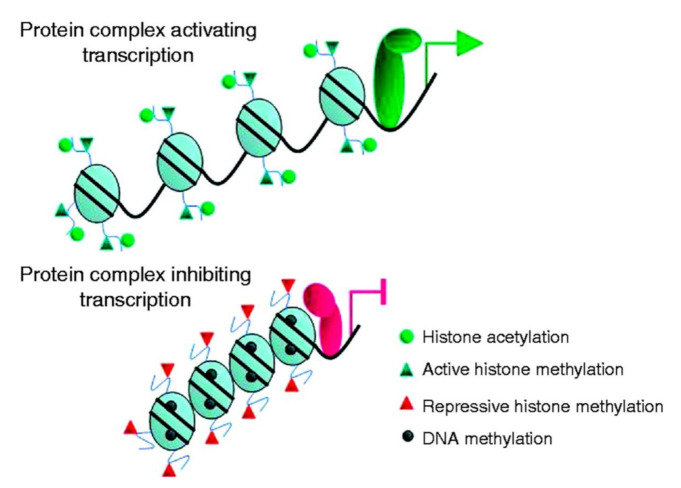
The (**upper**) panel illustrates euchromatin (activating transcription) and the (**lower**) panel heterochromatin (inhibiting transcription). The round green symbol illustrates histone acetylation; the blue triangle illustrates active histone methylation; the red triangle illustrates repressive histone methylation; the blue round symbol illustrates DNA methylation. Figure reprinted from Ref. [99], by permission.

Protein methylation (conjugation of a methyl group to a protein residue) is catalyzed by enzymes referred to as methyltransferases. The removal of the methyl group from the protein is regulated by demethylases. The most important role of methylation in cancer biology is to participate in epigenetic changes. Epigenetics, a phrase coined 80 years ago [98], are defined as genetic modifications that result in changes in the function and expression of genes without altering the DNA sequence. Epigenetic changes result in the altered structure of chromatin from an open configuration (euchromatin), permitting gene transcription, to a closed configuration (heterochromatin), representing tightly packed protein (histone)–DNA complexes that block gene transcription. The role of methylation in epigenetic changes is illustrated in Figure 3. The influence of these changes on thyroid tumorigenesis was reviewed recently by Russo et al. [99] and Rodriguez-Rodero et al. [100].

Methylation is involved in at least two steps of the epigenetic changes in tumor cells [99,100]. First, the methylation of one of the DNA bases (cytosine) is an important factor in the conversion of euchromatin to heterochromatin. Because cytosine-demethylases have not been identified, the cytosine methylation is a more stable epigenetic alteration than other epigenetic modifications. In cancer biology, DNA methylation in the promoter region of tumor-suppressor genes (silencing these genes) is particularly important [101,102].

The second mechanism through which methylation contributes to epigenetic changes is the posttranslational methylation of histones. The methylation of the *N*-terminal tail of histones occur at lysine and arginine residues and can result in mono-, di-, or tri-methylation of the histone protein [103,104]. The functional outcome of histone methylation depends on which residue is methylated and how many methyl groups are added to the protein; histone methylation can result in transcriptional activation (activating histone methylation) or transcriptional inactivation (repressive histone methylation). The activating histone methylation acts in parallel with histone acetylation, whereas the repressive histone methylation is accompanied by histone deacetylation. Adding to the complexity of histone modifications in epigenetic changes, histones can undergo posttranslational modifications through additional mechanisms, including phosphorylation, ubiquitination, sumoylation, O-GlcNAcylation, and ADP-ribosylation.

### Epigenetics and Thyroid Cancer

Epigenetic alterations have several important implications in thyroid tumorigenesis [105]. The silencing of several tumor suppressor genes by promoter hypermethylation and histone posttranslational methylation and deacetylation have been identified in thyroid cancer [106,107,108,109]. One of the tumor-suppressor genes silenced by promoter methylation is the phosphatase and tensin homologue (PTEN) gene [110]. Interestingly, the PTEN gene encodes a phosphatase that dephosphorylates PI3K and blocks the PI3K/Akt signaling pathway, providing a mechanism through which PTEN gene-promoter methylation induces a tumorigenic effect in the thyroid.

Epigenetic dysregulations also play a role in the insensitivity to radioiodine seen in poorly differentiated and anaplastic thyroid cancer [111]. Based on these and similar observations, demethylating agents have been proposed to restore the sensitivity to radioiodine treatment for certain aggressive thyroid cancers [111,112,113].

Understanding the role of methylation and other mechanisms involved in epigenetic changes in thyroid cancer is important not only because it provides an insight into thyroid tumorigenesis, but also because it can help develop diagnostic tools as well as novel treatments targeting the specific molecular mechanisms of thyroid malignancies.

## 7. Glycosylation

**Figure 4:**
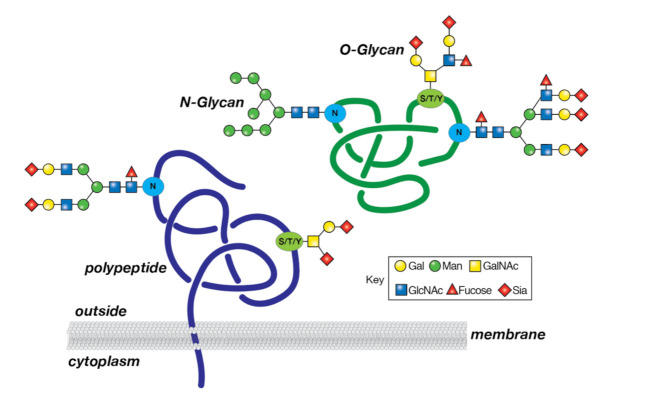
Glycoproteins are depicted containing Asn-linked (*N*-glycans) and Ser/Thr/Tyr-linked (S/T/Y) O-glycans. A membrane-bound glycoprotein is shown on the (**left**) and a soluble glycoprotein is on the (**right**). The monosaccharide residues (see the key) are added enzymatically to proteins in the endoplasmic reticulum (ER) and the Golgi apparatus by distinct enzymes.

Glycosylation is a post-translational process that involves the covalent assembly of monosaccharides to generate oligo- and polysaccharides (glycans) linked to proteins, resulting in the formation of glycoproteins (Figure 4). Glycoproteins arise through the secretory pathway involving the endoplasmic and Golgi apparatus, and then transit to the plasma membrane and other intracellular organelles. A common modification of secreted and membrane glycoproteins is the linkage of glycans to asparagine (*N*-glycosylation), which involves relatively large (>2 kDa) *N*-glycans that are typically branched, and rich in mannose, GlcNAc, galactose, fucose, and sialic acid. Another common modification is the addition of glycans to serine, threonine, and tyrosine (S/T/Y) O-glycosylation, and these O-glycans are generally smaller in size than the *N*-glycans. Glycosylation is regulated by specific enzymes in the endoplasmic reticulum/Golgi apparatus; these include glycosyltransferases that transfer monosaccharides, and glycohydrolases that remove monosaccharides.. For example, fucosyltransferases catalyze the addition of the monosaccharide fucose from the donor GDP-fucose to an acceptor polysaccharide chain, while sialyltransferases are responsible for the addition of sialic acid, using the donor CMP-sialic acid. Glycosylation is important to many cellular functions, as revealed by genetically heritable alterations in protein glycosylation. These alterations, termed congenital disorders of glycosylation (CDGs), of which there are >140 such disorders described to date, are associated with many development defects in humans, thus demonstrating the importance of protein glycosylation to normal homeostasis [114].

Evidence suggests that protein glycosylation is altered in many human cancers, including thyroid cancer. These alterations influence cell proliferation, migration, and differentiation, as well as the propensity for metastasis [115,116,117]. Significantly, altered glycosylation has led to the identification of sensitive biomarkers for cancer detection and surveillance. For example, fucosylated alpha-fetoprotein has proven to be a valuable marker for hepatocellular carcinoma, and CA 19-9, a fucosylated and sialylated carbohydrate antigen (Sialyl Lewis a/SLea) expressed on glycans in glycoproteins and glycolipids, is elevated in patients with colon and pancreatic cancer [118,119].

Glycomic profiling, the study of the entire population of glycans expressed in tissues or cells, allows for an understanding of the diverse nature of protein glycosylation in both normal and pathologic states, including malignancy [120]. The complexity of the human glycome and the characteristics of glycan expression, as well as the functional roles of glycoconjugates, have resulted in promising, yet challenging, opportunities for determining how these structures are involved in tumorigenesis and cancer progression. In the case of thyroid cancer, this complexity is further increased by different patterns of glycosylation in benign vs. malignant thyroid tumors and the involvement of specific enzymes in the tumorigenesis of different cancer subtypes.

### 7.1. Immunohistochemical Analysis Demonstrates Different Patterns of Sialylation and Fucosylation in Different Types of Thyroid Cancer

Previous immunohistochemical studies have demonstrated changes in glycosylation and implicated them in tumorigenesis of thyroid cancer. Nozawa et al. [121] utilized a monoclonal antibody to a sialic acid-dependent epitope, previously found to be correlated with invasion and metastasis of human tumors, on paraffin-embedded thyroid tissue samples. The results of these experiments demonstrated positive staining in nearly all FTCs (93%) and smaller proportions of PTCs (45%) and follicular adenomas (17%). By contrast, staining by FB21 was negative in adenomatous goiters, medullary carcinoma, and anaplastic carcinomas. It would be important in future studies to define the structure of the sialylated epitope recognized by FB21.

Another immunohistochemistry-based study by Ito et al. [122] characterized the expression of fucosyltransferase 8 (FUT8) in PTC. FUT8 catalyzes the addition of a fucose molecule to the GlcNAc linked to an asparagine residue, and in other studies, has been demonstrated to be highly expressed in hepatocellular and ovarian carcinomas [123,124]. The experiments by Ito et al. [122] demonstrated FUT8 expression in 33% of PTCs, but only 13% of FTCs. 

Taken together, the studies by Nozawa et al. [121] and Ito et al. [122] suggest that different patterns of glycosylation may be helpful in the diagnosis of different types of thyroid tumors. The functional role of these patterns in the tumorigenesis and cancer progression in thyroid neoplasms, however, has not yet been well elucidated.

### 7.2. Fucosylation of Epidermal Growth Factor Receptor (EGFR) by Fucosyltransferase 7 Is Involved in Cellular Proliferation and Migration in FTC

A recent study by Qin et al. [125] examined the expression of fucosyltransferase 7 (FUT7) in follicular thyroid cancers. In other studies, this enzyme, which catalyzes the addition of an α1,3-fucose molecule to a terminal GlcNAc residue to generate the sialyl Lewis x antigen (SLex), was implicated in tumor-cell proliferation, migration, and invasion in a number of human cancers [126,127]. The studies reported by Qin et al. [125] demonstrated increased FUT7 expression in follicular thyroid cancers compared to adjacent tissue by immunohistochemistry. The authors further examined these findings in different FTC cell lines, demonstrating the increased relative expression of FUT7 in a metastatic FTC cell line compared to a cell line derived from a primary FTC. In gain-of-function experiments, after the transfection of the primary FTC cell line with FUT7, the authors then demonstrated the upregulation of FUT7 expression, cell-cycle promotors, and in vitro proliferation and migration [125]. Furthermore, the authors demonstrated the increased α1,3-fucosylation of EGFR in FUT7-overexpressed cells, providing a potential mechanism of FUT7-mediated cellular proliferation and migration in FTC.

### 7.3. β1,6 GlcNAc Side Chain Branching of Matriptase N-Glycans by GNT-V Is Implicated in Early Malignant Transformation of PTC

Ito et al. [128] found that PTCs express *N*-acetylglucosaminyltransferase V (GNT-V), an enzyme responsible for the branching of complex *N*-glycans with β1,6 GlcNAc (*N*-acetylglucosamine). They proposed a role of GNT-V in malignant transformation. Further, the authors correlated GNT-V expression with the expression of matriptase, a serine protease that has been implicated in carcinoma metastasis, by immunohistochemistry, Western blotting, and polymerase chain-reaction techniques. Based on their observations, the authors concluded that the stabilization of matriptase via glycosylation by GNT-V may be involved in the early tumorigenesis of PTC.

### 7.4. Thyroglobulin as a Biomarker

Thyroglobulin (Tg) is a large glycoprotein (660 kD) produced by thyroid follicular cells and is involved in thyroid hormone production [129]. Tg contains 20 sites for *N*-glycosylation, of which 16 are known to be glycosylated under basal conditions [130]. Because serum concentrations of Tg may be elevated in both benign thyroid diseases and malignancy, its clinical utility in thyroid cancer has been under surveillance for the recurrence of malignancy after total thyroidectomy. After a total thyroidectomy, Tg falls to zero or very low levels; rising levels on follow-up may therefore be a sign of recurrent cancer.

Previous work has sought to characterize differences in the glycosylation of Tg in serum between patients with and without thyroid cancer. Shimizu et al. [131] utilized a lectin-based approach to characterize differences in the glycosylation of Tg in the sera of healthy patients and patients with benign or malignant thyroid tumors. Lectins are naturally occurring carbohydrate-binding proteins that have binding specificity to carbohydrate epitopes of glycans. For their study, the authors utilized Lens culinaris agglutinin (LCA), which is a lectin purified from the common lentil that binds to core fucosylated *N*-glycans, to quantify the ratio of LCA-reactive Tg to total Tg in patient sera. Among patients with high serum concentrations of thyroglobulin (>200 ng/mL), the ratios were significantly lower in those with cancer compared to those with benign nodules.

Kanai et al. [132] similarly analyzed the ratios of LCA-reactive Tg to total Tg from the sera of healthy individuals and patients with thyroid cancers utilizing an enzyme-linked immunofluorescence assay (ELISA) for the detection of Tg, which has a much higher sensitivity for the detection of Tg at low serum concentrations. This study demonstrated a reduction in the LCA-reactive-to-total-Tg ratio in patients with thyroid malignancies compared to healthy controls, regardless of total Tg serum concentration.

## 8. Succinylation

Succinylation is a recently identified PTM, involving the addition of a succinyl group to the lysine residue of a protein [133]. This PTM has been shown to be involved in alterations in cellular metabolism, suggesting that it plays a role in the coordination of metabolic pathways [134]. Additionally, histones are succinylated, adding to the complexity of ways in which PTMs may be involved in the regulation of gene expression [135]. While the mechanisms of succinylation are incompletely understood, recent evidence suggests it may occur by non-enzymatic mechanisms using succinyl-CoA as a cofactor [136,137], but there is also evidence of histone succinylation by KAT2A (GCN5) and histone acetyltransferase 1 (HAT1), acting as succinyltransferases [138,139].

Few studies have evaluated the role of succinylation in the promotion or inhibition of various human cancers [140]. Many previous studies of succinylation in the thyroid gland have evaluated the effect of succinylation on regulation of thyroid hormone synthesis and signaling [141,142]. The evaluation of succinylation in thyroid cancer to date is limited; however, it has been suggested that succinyl-CoA ligase subunit beta may be a useful protein biomarker for thyroid follicular carcinoma [143]. Additionally, mutations in the genes encoding succinate dehydrogenase (SDH), an enzyme involved in the electron transport chain and tricarboxylic acid cycle, have been shown to result in accumulation of succinate and succinyl-CoA [144]. Mutations in SDH have been identified in individuals with Cowden Syndrome; thus, it has been suggested that a role of succinylation in thyroid cancer may exist [140].

## 9. Summary and Conclusions

PTMs in thyroid cancer represent complex and diverse alterations with implications in cellular transformation and proliferation, tumorigenesis, invasion, and the development of metastases. Some of the PTMs, and their potential involvement in thyroid cancer, discussed in this review are briefly summarized in Table 1. These alterations and their downstream effects vary, depending on the enzymes involved, which proteins are being modified, and the cancer subtype. A better understanding of PTMs and their patterns in thyroid cancer will allow for an increased knowledge of tumor biology, the identification of novel biomarkers, and the improvement of treatment, with the development of individualized management of patients based on the determination of post-translationally modified proteins.

## Figures and Tables

**Table 1:** Summary of the proposed roles of posttranslational modifications in thyroid cancer.

Posttranslational Modification	Proposed Role in Thyroid Cancer
Ubiquitination	UBE4A-regulated ubiquitination and degradation of PCBP1 may be involved in the tumorigenesis of thyroid cancer. Thyroid cancer may be regulated by Smurf1-induced ubiquitination and the degradation of the tumor suppressor Kisspeptin-1.
Sumoylation	Reduced levels of sumoylated PDGF-C may be involved in tumorigenesis in thyroid cancer. Sumoylation of the tumor repressor CCDC6 may be a factor involved in thyroid cancer, providing an additional mechanism of sustained neoplastic growth. Sumoylation of TFAP2A results in altered patterns of gene expression associated with anaplastic thyroid cancer.
Acetylation	Histone acetylation varies by thyroid cancer subtype. Both histone acetyltransferase inhibitors and histone deacetylase inhibitors may have a role in the treatment of thyroid cancer.
Phosphorylation	Increased Akt phosphorylation and activity in thyroid cancer most often reflects stimulated PI3K-Akt signaling. CDK4/6-dependent phosphorylation of tumor suppressor RB may be important in the progression of anaplastic thyroid cancer.
Methylation	The PTEN tumor-suppressor gene is silenced by promotor methylation. PTEN gene promoter methylation induces a tumorigenic effect in the thyroid via dephosphorylation of PI3K.
Glycosylation	Sialic acid epitopes on glycoproteins are found in most follicular thyroid cancers (93%), but less than half (45%) of papillary thyroid cancers The stabilization of matriptase via glycosylation by GNT-V may be involved in the early tumorigenesis of PTC.
Succinylation	Succinyl-CoA ligase subunit beta may be a useful biomarker for follicular thyroid cancer. Mutations in succinate dehydrogenase in Cowden Syndrome may represent a role of succinylation in thyroid cancer.

## References

[1] Novelli F., Cappello P., Principe M., Bulfamante S. (2017). Alpha-Enolase i ENO1 i a potential target in novel immunotherapies. Front. Biosci..

[2] Yan F., Qian M., He Q., Zhu H., Yang B. (2020). The posttranslational modifications of Hippo-YAP pathway in cancer. Biochim. Et. Biophys. Acta (BBA)-Gen. Subj..

[3] Saji M., Ringel M.D. (2010). The PI3K-Akt-mTOR pathway in initiation and progression of thyroid tumors. Mol. Cell Endocrinol..

[4] Celano M., Mio C., Sponziello M., Verrienti A., Bulotta S., Durante C., Damante G., Russo D. (2018). Targeting post-translational histone modifications for the treatment of non-medullary thyroid cancer. Mol. Cell. Endocrinol..

[5] Li H., Tian Z., Qu Y., Yang Q., Guan H., Shi B., Ji M., Hou P. (2019). SIRT7 promotes thyroid tumorigenesis through phosphor-ylation and activation of Akt and p70S6K1 via DBC1/SIRT1 axis. Oncogene.

[6] Amit M., Na’ara S., Francis D., Matanis W., Zolotov S., Eisenhaber B., Eisenhaber F., Weiler Sagie M., Malkin L., Billan S. (2014). Post-translational regulation of radioactive iodine therapy response in papillary thyroid carcinoma. J. Mol. Endocrinol..

[7] Hałasa M., Wawruszak A., Przybyszewska A., Jaruga A., Guz M., Kałafut J., Stepulak A., Cybulski M. (2019). H3K18Ac as a Marker of Cancer Progression and Potential Target of Anti-Cancer Therapy. Cells.

[8] Miyoshi E., Ito Y., Miyoshi Y. (2010). Involvement of Aberrant Glycosylation in Thyroid Cancer. J. Oncol..

[9] Ciechanover A., Elias S., Heller H., Ferber S., Hershko A. (1980). Characterization of the heat-stable polypeptide of the ATP-dependent proteolytic system from reticulocytes. J. Biol. Chem..

[10] Cappadocia L., Lima C.D. (2017). Ubiquitin-like Protein Conjugation: Structures, Chemistry, and Mechanism. Chem. Rev..

[11] Popovic D., Vucic D., Dikic I. (2014). Ubiquitination in disease pathogenesis and treatment. Nat. Med..

[12] Pfoh R., Lacdao I.K., Saridakis V. (2015). Deubiquitinases and the new therapeutic opportunities offered to cancer. Endocr.-Relat. Cancer.

[13] Harper J.W., Ordureau A., Heo J.-M. (2018). Building and decoding ubiquitin chains for mitophagy. Nat. Rev. Mol. Cell Biol..

[14] Waxman S., Germain D. (2007). Targeting the Ubiquitin-Proteasome Pathway in Cancer Therapy. Anti-Cancer Agents Med. Chem..

[15] Datta K., Suman S., Kumar S., Fornacer A.J. (2016). Colorectal carcinogenesis, radiation quality, and the ubiquitin-proteasome path-way. J. Cancer.

[16] Liu J., Dong S., Wang H., Li L., Ye Q., Li Y., Miao J., Jhiang S., Zhao J., Zhao Y. (2019). Two distinct E3 ligases, SCF FBXL19 and HECW1, degrade thyroid transcription factor 1 in normal thyroid epithelial and follicular thyroid carcinoma cells, respectively. FASEB J..

[17] Wang H., Vardy L.A., Tan C.P., Loo J.M., Guo K., Li J., Lim S.G., Zhou J., Chng W.J., Ng S.B. (2010). PCBP1 Suppresses the Translation of Metastasis-Associated PRL-3 Phosphatase. Cancer Cell.

[18] Cho S.-J., Jung Y.-S., Chen X. (2013). Poly (C)-Binding Protein 1 Regulates p63 Expression through mRNA Stability. PLoS ONE.

[19] Zhang Z.-Z., Shen Z.-Y., Shen Y.-Y., Zhao E.-H., Wang M., Wang C.-J., Cao H., Xu J. (2015). HOTAIR Long Noncoding RNA Promotes Gastric Cancer Metastasis through Suppression of Poly r(C)-Binding Protein (PCBP) 1. Mol. Cancer Ther..

[20] Zhang M.-P., Zhang W.-S., Tan J., Zhao M.-H., Lian L.-J., Cai J. (2017). Poly r(C) binding protein (PCBP) 1 expression is regulated at the post-translation level in thyroid carcinoma. Am. J. Transl. Res..

[21] Zhang M.-P., Zhang W.-S., Tan J., Zhao M.-H., Lian L.-J., Cai J. (2017). Poly r(C) binding protein (PCBP) 1 expression is regulated by the E3 ligase UBE4A in thyroid carcinoma. Biosci. Rep..

[22] Yan C., Su H., Song X., Cao H., Kong L., Cui W. (2018). Smad Ubiquitination Regulatory Factor 1 (Smurf1) Promotes Thyroid Cancer Cell Proliferation and Migration via Ubiquitin-Dependent Degradation of Kisspeptin-1. Cell. Physiol. Biochem..

[23] Ji K., Ye L., Mason M.D., Jiang W.G. (2013). The Kiss-1/Kiss-1R complex as a negative regulator of cell motility and cancer metastasis (Review). Int. J. Mol. Med..

[24] Savvidis C., Papaoiconomou E., Petraki C., Msaouel P., Koutsilieris M. (2015). The role of KISS1/KISS1R system in tumor growth and invasion of differentiated thyroid cancer. Anticancer Res..

[25] Gao R.J., Wang N.P., Hui Y.E., Gao Q.J., Zhou Y., Duan H.S. (2010). Expression and clinical significance of tumor suppressor gene Kiss-1 in papillary thyroid carcinoma. Chin. J. Bases Clin. Gen. Surg..

[26] Bergers G., Benjamin L.E. (2003). Tumorigenesis and the angiogenic switch. Nat. Cancer.

[27] Dvorak H.F. (2003). Rous-Whipple Award Lecture. How Tumors Make Bad Blood Vessels and Stroma. Am. J. Pathol..

[28] Bunone G., Vigneri P., Mariani L., But S., Collini P., Pilotti S., Pierotti M.A., Bongarzone I. (1999). Expression of angiogenesis stimula-tors and inhibitors in human thyroid tumors and correlation with clinical pathological features. Am. J. Pathol..

[29] Vieira J., Santos S.C.R., Espadinha C., Correia I., Vag T., Casalou C., Cavaco B., Catarino A.L., Dias S., Leite V. (2005). Expression of vascular endothelial growth factor (VEGF) and its receptors in thyroid carcinomas of follicular origin: A potential autocrine loop. Eur. J. Endocrinol..

[30] Rodriguez-Antona C., Pallares J., Montero-Conde C., Inglada-Pérez L., Castelblanco E., Landa I., Leskelä S., Leandro-García L.J., López-Jiménez E., Letón R. (2010). Overexpression and activation of EGFR and VEGFR2 in medullary thyroid carcinomas is related to metastasis. Endocr. Relat. Cancer.

[31] Shaik S., Nucera C., Inuzuka H., Gao D., Garnaas M., Frechette G., Harris L., Wan L., Fukushima H., Husain A. (2012). SCF(β-TRCP) suppresses angiogenesis and thyroid cancer cell migra-tion by promoting ubiquitination and destruction of VEGF receptor 2. J. Exp. Med..

[32] Gupta-Abramson V., Troxel A., Nellore A., Puttaswamy K., Redlinger M., Ransone K., Mandel S.J., Flaherty K.T., Loevner L.A., O’Dwyer P.J. (2008). Phase II Trial of Sorafenib in Advanced Thyroid Cancer. J. Clin. Oncol..

[33] Cabanillas M.E., Waguespack S.G., Bronstein Y., Williams M.D., Feng L., Hernandez M., Lopez A., Sherman S.I., Busaidy N.L. (2010). Treatment with Tyrosine Kinase Inhibitors for Patients with Differentiated Thyroid Cancer: The, M.D. Anderson Experience. J. Clin. Endocrinol. Metab..

[34] Nikiforov Y.E., Seethala R.R., Tallini G., Baloch Z.W., Basolo F., Thompson L.D., Barletta J.A., Wenig B.M., Al Ghuzlan A., Kakudo K. (2016). Nomenclature revision for encapsulated follicular variant of papillary thyroid carcinoma: A paradigm shift to reduce overtreatment of indolent tumors. JAMA Oncol..

[35] Lindeman B.M., Nehs M.A., Angell T.E., Alexander E.K., Gawande A.A., Moore F.D., Doherty G.M., Cho N.L. (2019). Effect of noninva-sive follicular thyroid neoplasm with papillary-like nuclear features (NIFTP) on malignancy rates in thyroid nodules: How to counsel patients on extent of surgery. Ann. Surg. Oncol..

[36] Higgins S., James B., Sacks B., Mowschenson P., Nishino M., Hasselgren P.O. (2021). Can cytologic and sonographic features guide ex-tent of surgery and prevent “overtreatment” of thyroid nodules classified as suspicious for malignancy (Bethesda V)?. J. Surg. Res..

[37] Brandler T.C., Zhou F., Liu C.Z., Cho M., Lau R.P., Simsir A., Patel K.N., Sun W. (2017). Can noninvasive follicular thyroid neoplasm with papillary-like nuclear features be distinguished from classic papillary thyroid carcinoma and follicular adenomas by fi-ne-needle aspiration?. Cancer Cytopathol..

[38] Strickland K.C., Eszlinger M., Paschke R., Angell T.E., Alexander E.K., Marqusee E., Nehs M.A., Jo V.Y., Lowe A., Vivero M. (2018). Molecular Testing of Nodules with a Suspicious or Malignant Cytologic Diagnosis in the Setting of Non-Invasive Follicular Thyroid Neoplasm with Papil-lary-Like Nuclear Features (NIFTP). Endocr. Pathol..

[39] Al-Brahim N., Asa S.L. (2006). Papillary thyroid carcinoma: An overview. Arch. Pathol. Lab. Med..

[40] Ip Y.T., Filho M.A.D., Chan J.K.C. (2010). Nuclear inclusions and pseudoinclusions: Friends or foes of the surgical pathologist?. Int. J. Surg. Pathol..

[41] Seethala R.R., Baloch Z.W., Barletta J.A., Khanafshar E., Mete O., Sadow P.M., LiVolsi V.A., Nikiforov Y.E., Tallini G., Thompson L. (2018). Noninvasive follicular thyroid neoplasm with papillary-like nuclear features: A review for pathologists. Mod. Pathol..

[42] Bizzarro T., Martini M., Capodimonti S., Straccia P., Lombardi C.P., Pontecorvi A., Larocca L.M., Rossi E.D. (2016). Young investigator challenge: The morphologic analysis of noninvasive follicular thyroid neoplasm with papillary-like nuclear features on liq-uid-based cytology: Some insights into their identification. Cancer Cytopathol..

[43] Howitt B.E., Chang S., Eszlinger M., Paschke R., Drage M.G., Krane J.F., Barletta J.A. (2015). Fine-needle aspiration diagnoses of noninva-sive follicular variant of papillary thyroid carcinoma. Am. J. Clin. Pathol..

[44] Cracolici V., Krausz T., Cipriani N.A. (2018). Ubiquitin Immunostaining in Thyroid Neoplasms Marks True Intranuclear Cytoplasmic Pseudoinclusions and May Help Differentiate Papillary Carcinoma from NIFTP. Head Neck Pathol..

[45] Inabnet W.B., Palazzo F., Sosa J.A., Kriger J., Aspinall S., Barczynski M., Doherty G., Iacobone M., Nordenstrom E., Scott-Coombes D. (2019). Correlating the Bethesda System for Reporting Thyroid Cytopathology with Histology and Extent of Surgery: A Review of 21,746 Patients from Four Endocrine Surgery Registries Across Two Continents. World J. Surg..

[46] Yang Y., He Y., Wang X., Liang Z., He G., Zhang P., Zhu H., Xu N., Liang S. (2017). Protein SUMOylation modification and its associa-tions with disease. Open Biol..

[47] Han Z.J., Feng Y.H., Gu B.H., Li Y.M., Chen H. (2018). The post-translational modification, SUMOylation, and cancer (Review). Int. J. Oncol..

[48] Reigstad L.J., Martinez A., Varhaug J.E., Lillehaug J.R. (2006). Nuclear localisation of endogenous SUMO-1-modified PDGF-C in human thyroid tissue and cell lines. Exp. Cell Res..

[49] De Andrade J.P., Lorenzen A.W., Wu V.T., Bogachek M.V., Park J.M., Gu V.W., Sevenich C.M., Cassady V.C., Beck A.C., Kulak M.V. (2017). Targeting the SUMO pathway as a novel treatment for anaplastic thyroid cancer. Oncotarget.

[50] Luise C., Merolla F., Leone V., Paladino S., Sarnataro D., Fusco A., Celetti A. (2012). Identification of Sumoylation Sites in CCDC6, the First Identified RET Partner Gene in Papillary Thyroid Carcinoma, Uncovers a Mode of Regulating CCDC6 Function on CREB1 Transcriptional Activity. PLoS ONE.

[51] Tuccilli C., Baldini E., Sorrenti S., Di Gioia C., Bosco D., Ascoli V., Mian C., Barollo S., Rendina R., Coccaro C. (2015). Papillary thyroid cancer is characterized by altered expression of gens involed in the sumoylation process. J. Biol. Regul. Homeost. Agents.

[52] Li X., Pontén A., Aase K., Karlsson L., Abramsson A., Uutela M., Bäckström G., Hellström M., Boström H., Li H. (2000). PDGF-C is a new protease-activated ligand for the PDGF al-pha-receptor. Nat. Cell Biol..

[53] Li X., Eriksson U. (2003). Novel PDGF family members: PDGF-C and PDGF-D. Cytokine Growth Factor Rev..

[54] Leone V., Mansueto G., Pierantoni G.M., Tornincasa M., Merolla F., Cerrato A., Santoro M., Grieco M., Scaloni A., Celetti A. (2010). CCDC6 represses CREB1 activity by recruiting histone deacetylase 1 and protein phosphatase 1. Oncogene.

[55] Are C., Shaha A.R. (2006). Anaplastic Thyroid Carcinoma: Biology, Pathogenesis, Prognostic Factors, and Treatment Approaches. Ann. Surg. Oncol..

[56] Watson J.D., Baker T.A., Gann A., Levine M., Losik R. (2014). Molecular Biology of the Gene.

[57] Verdone L., Agricola E., Caserta M., Di Mauro E. (2006). Histone acetylation in gene regulation. Brief Funct. Genom. Proteomic.

[58] Glozak M.A., Sengupta N., Zhang X., Seto E. (2005). Acetylation and deacetylation of non-histone proteins. Gene.

[59] Yang X.-J., Seto E. (2008). Lysine Acetylation: Codified Crosstalk with Other Posttranslational Modifications. Mol. Cell.

[60] De Ruijter A.J.M., Van Gennip A.H., Caron H.N., Kemp S., Van Kuilenburg A.B.P. (2003). Histone deacetylases (HDACs): Characterization of the classical HDAC family. Biochem. J..

[61] Barnes P.J., Adcock I., Ito K. (2005). Histone acetylation and deacetylation: Importance in inflammatory lung diseases. Eur. Respir. J..

[62] Cao D.J., Wang Z.V., Battiprolu P.K., Jiang N., Morales C.R., Kong Y., Rothermel B.A., Gillette T.G., Hill J.A. (2011). Histone deacetylase (HDAC) inhibitors attenuate cardiac hypertrophy by suppressing autophagy. Proc. Natl. Acad. Sci. USA.

[63] Lorenz H., Lehmann L.H., Worst B.C., Stanmore D.A., Backs J. (2014). Histone deacetylase signaling in cardioprotection. Cell Mol. Life Sci..

[64] Hasselgren P.O. (2007). Ubiquitination, phosphorylation, and acetylation--triple threat in muscle wasting. J. Cell Physiol..

[65] Alamdari N., Aversa Z., Castillero E., Hasselgren P.-O. (2012). Acetylation and deacetylation—novel factors in muscle wasting. Metabolism.

[66] Lee J., Hwang Y.J., Kim K.Y., Kowall N.W., Ryu H. (2013). Epigenetic Mechanisms of Neurodegeneration in Huntington’s Disease. Neurotherapeutics.

[67] Glozak M.A., Seto E. (2007). Histone deacetylases and cancer. Oncogene.

[68] Cohen I., Porƒôba E., Kamieniarz K., Schneider R. (2011). Histone modifiers in cancer: Friends or foes?. Genes Cancer.

[69] Puppin C., Passon N., Lavarone E., Di Loreto C., Frasca F., Vella V., Vigneri R., Damante G. (2011). Levels of histone acetylation in thyroid tumors. Biochem. Biophys. Res. Commun..

[70] Russo D., Durante C., Bulotta S., Puppin C., Puxeddu E., Filetti S., Damante G. (2012). Targeting histone deacetylase in thyroid cancer. Expert Opin. Ther. Targets.

[71] Lin C.L., Tsai M.L., Lin C.Y., Hsu K.W., Hsieh W.S., Chi W.M., Huang L.C., Lee C.H. (2019). HDAC1 and HDAC2 Double Knockout Trig-gers Cell Apoptosis in Advanced Thyroid Cancer. Int. J. Mol. Sci..

[72] Barneda-Zahonero B., Parra M. (2012). Histone deacetylases and cancer. Mol. Oncol..

[73] Manzo F., Tambaro F.P., Mai A., Altucci L. (2009). Histone acetyltransferase inhibitors and preclinical studies. Expert Opin. Ther. Patents.

[74] Cohen P. (2000). The regulation of protein function by multisite phosphorylation–a 25 year update. Trends Biochem. Sci..

[75] Singh V., Ram M., Kumar R., Prasad R., Roy B.K., Singh K.K. (2017). Phosphorylation: Implications in Cancer. J. Protein Chem..

[76] Hanahan D., Weinberg R.A. (2000). The hallmarks of cancer. Cell.

[77] Hunter T. (1995). Protein kinases and phosphatases: The Yin and Yang of protein phosphorylation and signaling. Cell.

[78] Ma J., Wu C., Hart G.W. (2021). Analytical and biochemical perspectives of protein O-GlcNAcylation. Chem. Rev..

[79] Xing M. (2013). Molecular pathogenesis and mechanisms of thyroid cancer. Nat. Cancer.

[80] Xing M. (2010). Genetic Alterations in the Phosphatidylinositol-3 Kinase/Akt Pathway in Thyroid Cancer. Thyroid.

[81] Cabanillas M.E., Ryder M., Jimenez C. (2019). Targeted Therapy for Advanced Thyroid Cancer: Kinase Inhibitors and Beyond. Endocr. Rev..

[82] Li Y., Yang Q., Guan H., Shi B., Ji M., Hou P. (2018). ZNF677 Suppresses Akt Phosphorylation and Tumorigenesis in Thyroid Cancer. Cancer Res..

[83] Jen J., Wang Y.C. (2016). Zinc finger proteins in cancer progression. J. Biomed. Sci..

[84] Mahajan K., Mahajan N.P. (2012). PI3K-independent AKT activation in cancers: A treasure trove for novel therapeutics. J. Cell. Physiol..

[85] Zhang P., Wang C., Ma T., You S. (2015). O-GlcNAcylation enhances the invasion of thyroid anaplastic cancer cells partially by PI3K/Akt1 pathway. Onco. Targets Ther..

[86] Krześlak A., Jóźwiak P., Lipińska A. (2011). Down-regulation of β-N-acetyl-D-glucosaminidase increases akt1 activity in thyroid an-aplastic cancer cells. Oncol. Rep..

[87] Cheng Y.U., Li H., Li J., Li J., Gao Y., Liu B. (2016). O-GlcNAcylation enhances anaplastic thyroid carcinoma malignancy. Oncol. Lett..

[88] Li X., Wu Z., He J., Jin Y., Chu C., Cao Y., Gu F., Wang H., Hou C., Liu X. (2021). OGT regulated O-GlcNAcylation promotes pa-pillary thyroid cancer malignancy via activating YAP. Oncogene.

[89] de Nigris F., Cerutti J., Morelli C., Califano D., Chiariotti L., Viglietto G., Santelli G., Fusco A. (2002). Isolation of a SIR-like gene, SIR-T8, that is overexpressed in thyroid carcinoma cell lines and tissues. Br. J. Cancer.

[90] Frye R. (2002). “SIRT8” expressed in thyroid cancer is actually SIRT7. Br. J. Cancer.

[91] Petrulea M.S., Plantinga T., Smit J.W., Georgescu C.E., Netea-Maier R.T. (2015). PI3K/Akt/mTOR: A promising therapeutic target for non-medullary thyroid carcinoma. Cancer Treat. Rev..

[92] Prasongsook N., Kumar A., Chintakuntlawar A., Foote R.L., Kasperbauer J., Molina J., Garces Y., Ma D., Wittich M.A.N., Rubin J. (2017). Survival in Response to Multimodal Therapy in Anaplastic Thyroid Cancer. J. Clin. Endocrinol. Metab..

[93] Saini S., Tulla K., Maker A.V., Burman K.D., Prabhakar B.S. (2018). Therapeutic advances in anaplastic thyroid cancer: A current per-spective. Mol. Cancer..

[94] Wong K., Di Cristofano F., Ranieri M., De Martino D., Di Cristofano A. (2019). PI3K/mTOR inhibition potentiates and extends palbo-ciclib activity in anaplastic thyroid cancer. Endocr. Relat. Cancer.

[95] Sherr C.J. (2000). The Pezcoller lecture: Cancer cell cycles revisited. Cancer Res..

[96] Lee H.J., Lee W.K., Kang C.W., Ku C.R., Cho Y.H., Lee E.J. (2018). A selective cyclin-dependent kinase 4, 6 dual inhibitor, Ribociclib (LEE011) inhibits cell proliferation and induces apoptosis in aggressive thyroid cancer. Cancer Lett..

[97] O’Leary B., Cutts R.J., Liu Y., Hrebien S., Huang X., Fenwick K., André F., Loibl S., Loi S., Garcia-Murillas I. (2018). The Genetic Landscape and Clonal Evolution of Breast Cancer Resistance to Palbociclib plus Fulvestrant in the PALOMA-3 Trial. Cancer Discov..

[98] Waddington C.H. (2012). The epigenotype. 1942. Int. J. Epidemiol..

[99] Russo D., Damante G., Puxeddu E., Durante C., Filetti S. (2011). Epigenetics of thyroid cancer and novel therapeutic targets. J. Mol. Endocrinol..

[100] Rodríguez-Rodero S., Delgado-Álvarez E., Fernández A.F., Fernández-Morera J.L., Menéndez-Torre E., Fraga M.F. (2014). Epigenetic alterations in endocrine-related cancer. Endocr.-Relat. Cancer.

[101] Kass S.U., Pruss D., Wolffe A.P. (1997). How does DNA methylation repress transcription?. Trends Genet..

[102] Baylin S.B., Herman J.G. (2000). DNA hypermethylation in tumorigenesis: Epigenetics joins genetics. Trends Genet..

[103] Kouzarides T. (2007). Chromatin modifications and their function. Cell.

[104] Zhang X., Wen H., Shi X. (2012). Lysine methylation: Beyond histones. Acta Biochim. Biophys Sin..

[105] Xing M. (2007). Gene Methylation in Thyroid Tumorigenesis. Endocrinology.

[106] Xing M., Usadel H., Cohen Y., Tokumaru Y., Guo Z., Westra W.B., Tong B.C., Tallini G., Udelsman R., Califano J.A. (2003). Methylation of the thyroid-stimulating hormone receptor gene in epithelial thyroid tumors: A marker of malig-nancy and a cause of gene silencing. Cancer Res..

[107] Hu S., Liu D., Tufano R.P., Carson K.A., Rosenbaum E., Cohen Y., Holt E.H., Kiseljak-Vassiliades K., Rhoden K.J., Tolaney S. (2006). Association of aberrant meth-ylation of tumor suppressor genes with tumor aggressiveness and BRAF mutation in papillary thyroid cancer. Int. J. Cancer.

[108] Kondo T., Nakazawa T., Ma D., Niu D., Mochizuki K., Kawasaki T., Nakamura N., Yamane T., Kobayashi M., Katoh R. (2009). Epige-netic silencing of TTF-1/NKX2-1 through DNA hypermethylation and histone H3 modulation in thyroid carcinomas. Lab-Vest.

[109] Zuo H., Gandhi M., Edreira M.M., Hochbaum D., Nimgaonkar V.L., Zhang P., Dipaola J., Evdokimova V., Altschuler D.L., Nikifo-rov Y.E. (2010). Downregulation of Rap1GAP through epigenetic silencing and loss of heterozygosity promotes invasion and progression of thyroid tumors. Cancer Res..

[110] Tell G., Pines A., Arturi F., Cesaratto L., Adamson E., Puppin C., Presta I., Russo D., Filetti S., Damante G. (2004). Control of Phosphatase and Tensin Homolog (PTEN) Gene Expression in Normal and Neoplastic Thyroid Cells. Endocrinology.

[111] Schlumberger M., Lacroix L., Russo D., Filetti S., Bidart J.-M. (2007). Defects in iodide metabolism in thyroid cancer and implications for the follow-up and treatment of patients. Nat. Clin. Pract. Endocrinol. Metab..

[112] Venkataraman G.M., Yatin M., Marcinek R., Ain K.B. (1999). Restoration of iodide uptake in dedifferentiated thyroid carcinoma: Rela-tionship to human Na+/I-symporter gene methylation status. J. Clin. Endocrinol. Metab..

[113] Haugen B.R. (2004). Redifferentiation therapy in advanced thyroid cancer. Curr. Drug Targets Immune Endocr Metab. Disord.

[114] Wilson M.P., Matthijs G. (2021). The evolving genetic landscape of congenital disorders of glycosylation. Biochim. Et. Biophys. Acta (BBA)-Gen. Subj..

[115] Fuster M.M., Esko J.D. (2005). The Sweet and Sour of Cancer: Glycans as Novel Therapeutic Targets. Nat. Rev. Cancer.

[116] Pinho S.S., Reis C.A. (2015). Glycosylation in cancer: Mechanisms and clinical implications. Nat. Rev. Cancer.

[117] Stowell S.R., Ju T., Cummings R.D. (2015). Protein glycosylation in cancer. Annu. Rev. Pathol. Mech. Dis..

[118] Aoyagi Y., Suzuki Y., Igarashi K. (1991). The usefulness of simultaneous determinations of glucosaminylation and fucosylation indices of alpha‚ Äêfetoprotein in the differential diagnosis of neoplastic diseases of the liver. Cancer.

[119] Taketa K., Endo Y., Sekiya C. (1993). A Collaborative Study for the Evaluation of Lectin-Reactive α-Fetoproteins in Early Detec-tion of Hepatocellular Carcinoma. Cancer Res..

[120] Cummings R.D., Pierce J.M. (2014). The Challenge and Promise of Glycomics. Chem. Biol..

[121] Nozawa Y., Ami H., Suzuki S., Tuchiya A., Abe R., Abe M. (1999). Distribution of sialic acid-dependent carbohydrate epitope in thy-roid tumors: Immunoreactivity of FB21 in paraffin-embedded tissue sections. Pathol. Int..

[122] Ito Y., Miyauchi A., Yoshida H., Uruno T., Nakano K., Takamura Y., Miya A., Kobayashi K., Yokozawa T., Matsuzuka F. (2003). Expression of α1,6-fucosyltransferase (FUT8) in papillary carcinoma of the thyroid: Its linkage to biological aggressiveness and anaplastic transformation. Cancer Lett..

[123] Noda K., Miyoshi E., Uozumi N., Yanagidani S., Ikeda Y., Gao C.-X., Suzuki K., Yoshihara H., Yoshikawa M., Kawano K. (1998). Gene expression of fucosyltransferase in human hepatoma tissues: A possible implication for increased fucosylation of fetoprotein. Hepatology.

[124] Takahashi T., Ikeda Y., Miyoshi E., Yagiimura Y., Ishikawa M., Taniguchi N. (2000). a1, 6 fucosyltransferase is highly and specifi-cally expressed in human ovarian serous adenocarcinomas. Int. J. Cancer.

[125] Qin H., Liu J., Yu M., Wang H., Thomas A.M., Li S., Yan Q., Wang L. (2020). FUT7 promotes the malignant transformation of follicular thyroid carcinoma through α-1,3-fucosylation of EGF receptor. Exp. Cell Res..

[126] Martin-Satue M., de Castellarnau C., Blanco J. (1999). Overexpression of alpha(1,3)-fucosyltransferase VII is sufficient for the ac-quisition of lung colonization phenotype in human lung adenocarcinoma HAL-24Luc cells. Br. J. Canc..

[127] Liu F., Qi H.L., Zhang Y., Zhang X.Y., Chen H.L. (2001). Transfection of the c-erbB2/neu gene upregulates the expression of sialyl Lewis X, alpha1,3-fucosyltransferase VII, and metastatic potential in a human hepatocarcinoma cell line. Eur. J. Biochem.

[128] Ito Y., Akinaga A., Yamanaka K., Nakagawa T., Kondo A., Dickson R.B., Lin C.-Y., Miyauchi A., Taniguchi N., Miyoshi E. (2006). Co-expression of matriptase and N-acetylglucosaminyltransferase V in thyroid cancer tissues—its possible role in prolonged stability in vivo by aberrant glycosylation. Glycobiology.

[129] Citterio C.E., Targovnik H.M., Arvan P. (2019). The role of thyroglobulin in thyroid hormonogenesis. Nat. Rev. Endocrinol..

[130] Yang S.-X., Pollock H., Rawitch A.B. (1996). Glycosylation in Human Thyroglobulin: Location of the N-Linked Oligosaccharide Units and Comparison with Bovine Thyroglobulin. Arch. Biochem. Biophys..

[131] Shimizu K., Nakamura K., Kobatake S., Satomura S., Maruyama M., Kameko F., Tajiri J., Kato R. (2007). The clinical utility of Lens culinaris agglutinin-reactive thyroglobulin ratio in serum for distinguishing benign from malignant conditions of the thyroid. Clin. Chim. Acta.

[132] Kanai T., Amakawa M., Kato R., Shimizu K., Nakamura K., Ito K.-I., Hama Y., Fujimori M., Amano J. (2009). Evaluation of a new method for the diagnosis of alterations of Lens culinaris agglutinin binding of thyroglobulin molecules in thyroid carcinoma. Clin. Chem. Lab. Med. (CCLM).

[133] Zhang Z., Tan M., Xie Z., Dai L., Chen Y., Zhao T. (2011). Identification of lysine succinylation as a new post-translational modifica-tion. Nat. Chem. Biol..

[134] Yang Y., Gibson G.E. (2019). Succinylation Links Metabolism to Protein Functions. Neurochem. Res..

[135] Xie Z., Dai J., Dai L., Tan M., Cheng Z., Wu Y., Boeke J., Zhao Y. (2012). Lysine Succinylation and Lysine Malonylation in Histones. Mol. Cell. Proteom..

[136] Simithy J., Sidoli S., Yuan Z.-F., Coradin M., Bhanu N.V., Marchione D., Klein B.J., Bazilevsky G.A., McCullough C.E., Magin R. (2017). Characterization of histone acylations links chromatin modifications with metabolism. Nat. Commun..

[137] Wagner G.R., Bhatt D.P., O’Connell T.M., Thompson J.W., Dubois L.G., Backos D.S., Yang H., Mitchell G.A., Ilkayeva O.R., Stevens R.D. (2017). A class of reactive acyl-CoA species reveals the non-enzymatic origins of protein acylation. Cell Metab..

[138] Wang Y., Guo Y.R., Liu K., Yin Z., Liu R., Xia Y., Tan L., Yang P., Lee J.H., Li X.J. (2017). KAT2A coupled with the α-KGDH complex acts as a histone H3 succinyltransferase. Nature.

[139] Yang G., Yuan Y., Yuan H., Wang J., Yun H., Geng Y., Zhao M., Li L., Weng Y., Liu Z. (2021). Histone acetyltransferase 1 is a suc-cinyltransferase for histones and non-histones and promotes tumorigenesis. EMBO Rep..

[140] Mu R., Ma Z., Lu C., Wang H., Cheng X., Tuo B., Fan Y., Liu X., Taolang L. (2021). Role of succinylation modification in thyroid cancer and breast cancer. Am. J. Cancer Res..

[141] Dunn J., Kim P., Dunn A. (1982). Favored sites for thyroid hormone formation on the peptide chains of human thyroglobulin. J. Biol. Chem..

[142] Shifrin S., Kohn L.D. (1981). Binding of thyroglobulin to bovine thyroid membranes. Role of specific amino acids in receptor recogni-tion. J. Biol. Chem..

[143] Lai X., Umbricht C.B., Fisher K., Bishop J., Shi Q., Chen S. (2017). Identification of novel biomarker and therapeutic target candidates for diagnosis and treatment of follicular carcinoma. J. Proteom..

[144] Smestad J., Erber L., Chen Y., Maher L.J. (2018). Chromatin Succinylation Correlates with Active Gene Expression and Is Perturbed by Defective TCA Cycle Metabolism. iScience.

